# Treating depression and improving adherence in HIV care with task‐shared cognitive behavioural therapy in Khayelitsha, South Africa: a randomized controlled trial

**DOI:** 10.1002/jia2.25823

**Published:** 2021-10-28

**Authors:** Steven A. Safren, Conall O'Cleirigh, Lena S. Andersen, Jessica F. Magidson, Jasper S. Lee, Sierra A. Bainter, Nicholas Musinguzi, Jane Simoni, Ashraf Kagee, John A. Joska

**Affiliations:** ^1^ Department of Psychology University of Miami Miami Florida USA; ^2^ Department of Psychology Harvard Medical School Boston Massachusetts USA; ^3^ Department of Psychiatry Massachusetts General Hospital Boston Massachusetts USA; ^4^ Department of Psychiatry University of Cape Town Cape Town South Africa; ^5^ Department of Psychology University of Maryland College Park Maryland USA; ^6^ Department of Internal Medicine Mbarara University of Science and Technology Mbarara Uganda; ^7^ Department of Psychology University of Washington Seattle Washington USA; ^8^ Department of Psychology Stellenbosch University Stellenbosch South Africa

**Keywords:** HIV care continuum, ARV, adherence, intervention, depression, cognitive behavioural therapy (CBT), task sharing, task shifting, randomized controlled trial, global mental health

## Abstract

**Introduction:**

Major depressive disorder, highly prevalent among people with HIV (PWH) globally, including South Africa, is associated with suboptimal adherence to antiretroviral therapy. Globally, there are insufficient numbers of mental health providers and tested depression treatments. This study's aim was to test task‐shared cognitive‐behavioural therapy for adherence and depression (CBT‐AD) in HIV, delivered by clinic nurses in South Africa.

**Methods:**

This was a two‐arm randomized controlled effectiveness trial (recruitment: 14 July 2016 to 4 June 2019, last follow 9 June 2020). One‐hundred‐sixty‐one participants with clinical depression and virally uncontrolled HIV were recruited from primary care clinics providing HIV care, in Khayelitsha, South Africa. Arm 1 was task‐shared, nurse‐delivered CBT‐AD; and arm 2 was enhanced treatment as usual (ETAU). Primary outcomes (baseline to 4 months) were blinded Hamilton Depression Rating Scale (HAM‐D) scores, and weekly adherence via real‐time monitoring (Wisepill). Secondary outcomes were adherence and depression over 4‐, 8‐ and 12‐month follow‐ups, proportion of participants with undetectable viremia and continuous CD4 cell counts at 12 months. Additional analyses involved viral load and CD4 over time.

**Results:**

At 4 months, the HAMD scores in the CBT‐AD condition improved by an estimated 4.88 points more (CI: –7.86, –1.87, *p* = 0.0016), and for weekly adherence, 1.61 percentage points more per week (CI: 0.64, 2.58, *p* = 0.001) than ETAU. Over follow‐ups, CBT‐AD had an estimated 5.63 lower HAMD scores (CI: –7.90, –3.36, *p* < 0.001) and 23.56 percentage points higher adherence (CI: 10.51, 34.21, *p* < 0.001) than ETAU. At 12 months, adjusted models indicated that the odds of having an undetectable viremia was 2.51 greater at 12 months (CI: 1.01, 6.66, *p* = 0.047), and 3.54 greater over all of the follow‐ups (aOR = 3.54, CI: 1.59, 20.50; *p* = 0.038) for those assigned CBT‐AD. CD4 was not significantly different between groups at 12 months or over time.

**Conclusions:**

Task‐shared, nurse‐delivered, CBT‐AD is effective in improving clinical depression, ART adherence and viral load for virally unsuppressed PWH. The strategy of reducing depression to allow patients with self‐care components of medical illness to benefit from adherence interventions is one to extend. Implementation science trials and analyses of cost‐effectiveness are needed to translate findings into clinical practice.

**Trial Registration:**

ClinicalTrials.gov Identifier: NCT02696824 https://clinicaltrials.gov/ct2/show/NCT02696824

## 1 INTRODUCTION

South Africa is the home to the highest number of HIV/AIDS cases globally [1]. Like other settings, clinical depression is a prevalent and impairing comorbid illness. In one study of 900 people living with HIV (PLWH) across 18 recruitment sites and 5 provinces in South Africa, the prevalence of major depression was 11.1% and mild depression 29.9% [2]. Among PLWH, depression is consistently associated with poor adherence to antiretroviral therapy (ART) [3], and thus potentially viral detectability and poor long‐term outcomes. In 2018, only approximately 54% of HIV‐infected individuals in South Africa were on ART and virally suppressed [1]. Additionally, only approximately 10–25% of individuals in South Africa with a common mental disorder receive treatment [4, 5]. Given the prevalence of depression and its association with poor adherence, not only in HIV [3] but across medical conditions [6], it is important to treat depression in HIV for quality of life improvements and HIV control [7].

One barrier to treating depression for PLWH in resource‐constrained settings is the lack of mental health professionals available [8] — only 0.28 psychiatrists and 0.32 psychologists per 100,000 people in South Africa [9]. The strategy of task sharing, formerly termed task “shifting” [10], is, therefore, needed to address the mental health needs of PLWH [11].

Cognitive behavioural therapy (CBT) is a well‐validated treatment of depression that has previously been adapted for low‐resource settings and PLWH [12]. Given its structure and brevity, and the feasibility of training non‐specialists in CBT, it is a good candidate for task sharing in this context. While prospective medication trials for depression in HIV have not shown effects on ART adherence [13, 14, 15], integrating CBT for depression with CBT for adherence (CBT‐AD) has shown improvements in adherence [12, 16, 17, 18]. In these trials, CBT‐AD integrated the Life‐Steps adherence counselling intervention [19, 20] throughout 8–10 sessions of CBT for depression, delivered by psychologists or psychology trainees. To support task‐sharing CBT‐AD in the South African setting, we adapted the intervention for PLWH in South Africa, pilot tested it with nurse interventionists in a small open trial and had promising results on feasibility, acceptability and effectiveness outcomes [21]. Accordingly, the present trial aimed to test the effectiveness of this task‐shared approach to treating depression and adherence in HIV in a larger, randomized design in a South African peri‐urban HIV care setting.

## 2 METHODS

### 2.1 Trial design

This was a two‐arm randomized (1:1 ratio; detailed below) controlled effectiveness trial over a 12‐month period (protocol paper [22]). The two arms were (1) enhanced treatment as usual (ETAU) which all participants received, and (2) task‐shared CBT‐AD delivered by a trained nurse interventionist (hired by the study team, dedicated to the clinical and research duties involved in the trial) in addition to ETAU. The first study phase was the acute outcome (randomization to 4 months) when the treatment of depression and adherence with CBT‐AD was completed. The second phase included the 4‐, 8‐ and 12‐month follow‐up assessments. Randomization occurred approximately 4 weeks after baseline, allowing for stratification on potential antidepressant uptake.

All procedures were reviewed and approved by the Institutional Review Board at the University of Miami and the Human Research Ethics Committee at the University of Cape Town. All participants completed an informed consent process in their preferred language with a study clinician prior to undergoing any study procedures.

### 2.2 Participants

Included were PLWH who had a current diagnosis of depression and did not attain viral suppression from first‐line ART per local clinic standard (HIV RNA viral load > 400 copies/ml after first‐line treatment). Excluded were individuals who were unable or unwilling to provide informed consent, had active untreated major mental illness (e.g. untreated psychotic or mania) that would interfere with treatment, had received CBT for depression or who were <18 years old.

Assessment and treatment sessions took place at one of two public clinics that provide HIV services in Khayelitsha, a peri‐urban community outside of Cape Town. No psychological services are available at these clinics, although the medical officer can prescribe antidepressants and refer to a larger provincial clinic or an NGO (see ETAU). The period of study recruitment was 14 July 2016 to 4 June 2019, with the 12‐month follow‐up period extending until 9 June 2020.

Bilingual research assistants at the clinics identified potential participants in HIV care. Potential participants were screened using the major depressive disorder (MDD) module of the Mini International Neuropsychiatric Interview (MINI) [23]. Participants who met criteria for MDD who did not have a recent (past month) HIV viral load on file underwent a blood draw for HIV RNA viral load and CD4.

#### 2.2.1 Interventions

*CBT‐AD* was delivered at both study sites over eight sessions by trained nurses. We chose nurses given that they are consistently funded at clinics (compared to lay adherence counsellors historically funded by NGOs). The nurses had a mental health background but were not trained in psychotherapy before this project. Note, in South Africa, there are different sub‐cadres within nursing. Specialist mental health nurses are appointed into both in‐ and out‐patient mental health services. For the purposes of this study, we hired professional nurses with mental health training, as they are appointed in primary care settings. CBT‐AD implemented in this study was adapted based upon findings from our formative work [21], our experience providing training and clinical supervision to the nurses and input from multiple community advisory board meetings. Specifically, formative work pointed to cognitive restructuring being less feasible for task sharing and with this population due to training needs, patient comprehension and less cultural relevance. In addition, to facilitate task sharing, we adapted the original manual to a flipbook translated into the local language (isiXhosa). The flipbook provided reminders and tips for the nurse‐interventionist on one side and the “workbook” content for the participant to see on the other side, while allowing the interventionists to maintain eye contact. The CBT‐AD treatment was organized across five modules: (1) Life‐Steps adherence counselling [19, 20], adapted to the local setting [24]. Future sessions involved integrating Life‐Steps with modules for depression and HIV. These additional modules included (2) Introduction to CBT, psychoeducation about the nature of depression and motivational interviewing for behaviour change (≈ one session); (3) Behavioural activation: increasing value‐driven pleasurable activities and mood monitoring (≈ two sessions); (3) Problem‐solving, particularly for problems related to HIV self‐care (≈ two sessions); (4) Relaxation training (≈ one session) and (5) Review and relapse prevention (≈ one session). The nurse‐interventionist was able to structure the number of sessions and time spent on each module to meet individualized participants’ needs. The CBT skills were applied to support adaptive HIV disease management. Participants had the option to participate in up to 9 monthly booster sessions following treatment completion that included a brief review of content. Participants were reimbursed for travel but not compensated to attend sessions. Approximately two‐thirds (67.5%) of participants (*n* = 54) attended only one booster session, 51.25% (*n* = 41) attended two boosters, 38.75% (*n* = 31) attended three boosters and 35% (*n* = 28) attended four or more booster sessions. Additional details on the original intervention are published in treatment manuals, [25]; and additional procedures for intervention delivery/training are in the protocol paper [22]. Ten percent of sessions were randomly selected for fidelity rating: average session adherence was 94.54% (SD = 7.12%), and the average therapist competence was 82.23% (SD = 6.86%).

#### 2.2.2 Comparison condition: ETAU

Participants in both conditions received ETAU, including feedback to the participant and to their HIV care provider concerning participants’ diagnosis of depression. A provider letter stated that the participant's involvement in the study should not change how they would assess or treat depression, therefore, allowing for opportunity for the participants to be prescribed antidepressants. Usual care for those who had not achieved viral suppression included medications for 1 month and meeting with the adherence counsellor individually, a return visit and repeat prescription with adherence counselling, repeated again (3 months later) with blood draw and additional adherence counselling. If viral load is still >1000, the patient is referred to a risk of treatment failure group which entails, typically, additional adherence support such as one to three counselling sessions that may or may not be following a structured format (similar to Life‐Steps).

### 2.3 Outcomes and assessments

There were two a‐priori co‐primary outcomes: adherence, assessed via an electronic Wisepill device [26] between baseline and the 4‐month assessment, and via the Hamilton Depression Scale (HAM‐D; blinded assessor) [27] at the 4‐month assessment. Participants in both arms received compensation for time spent in the assessment visits. A‐priori secondary outcomes included depression as assessed by self‐report (Center for Epidemiologic Studies Depression Scale; CESD) [28] at acute and follow‐up, HAM‐D clinician‐assessed depression over follow‐up, adherence over follow‐up, the percentage of participants with undetectable HIV RNA at 12 months and CD4 cell counts at 12 months. Exploratory analyses were also conducted with HIV RNA viral load and CD4 cell counts over all timepoints.

#### 2.3.1 Depression

At each major visit (baseline, 4‐, 8‐ and 12‐month), a trained blinded assessor completed the HAM‐D [27, 28]. Assessors were trained by certified trainers and supervised on a weekly basis to prevent rating drift. Participants also completed the CESD [28] at each timepoint. See Appendix S2 for details on translation process.

#### 2.3.2 Adherence

We used real‐time electronic monitoring (Wisepill), which transmits a real‐time signal to a web‐server when the pillbox is opened. Participants’ weekly adherence was determined based on the number of times they opened the box divided by the number of times they were supposed to take pills.

#### 2.3.3 Biomedical outcomes

At baseline and follow‐ups, we extracted HIV viral load and CD4 cell data from patient medical records (see protocol paper for details about assays [22]). If participants did not have recent (1‐month) testing, we collected blood samples.

### 2.4 Sample size and power

**Figure 1:**
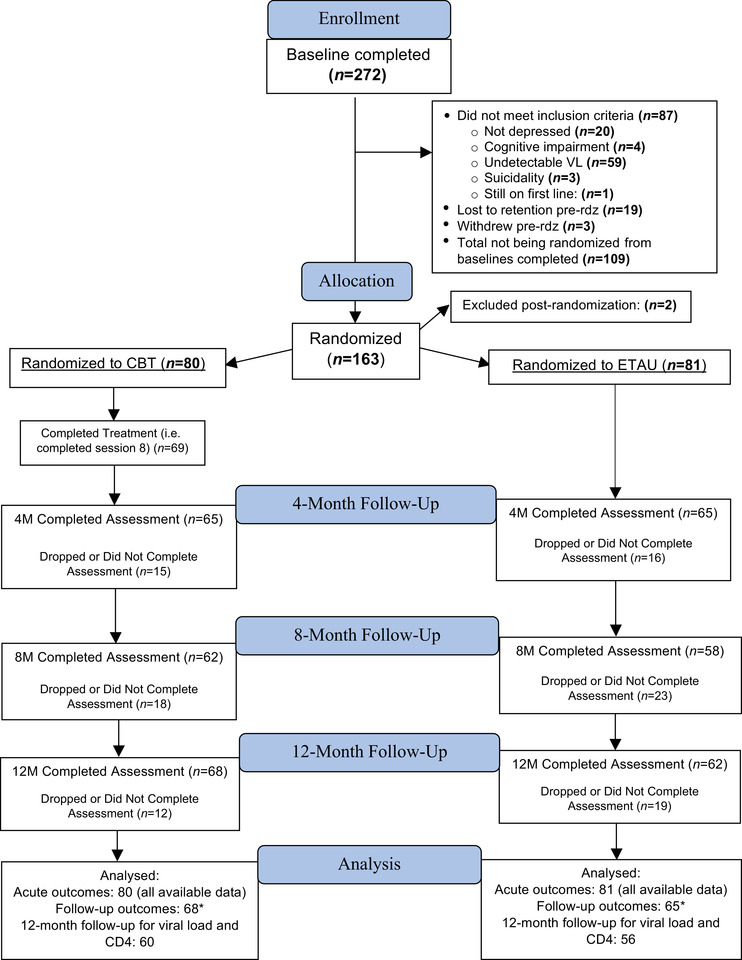
CONSORT diagram. Abbreviations: CBT, cognitive behavioral therapy for adherence and depression; ETAU, enhanced treatment as usual; pre‐rdz, pre‐randomization; 4M, 8M and 12M, 4‐, 8‐ and 12‐month, respectively. ^*^Follow‐up analyses used data for participants who attended any of the three follow‐up visits.

There were 161 individuals randomized and analysed in the study (Figure 1). Two participants were excluded after randomization occurred because of an issue related to the timing of viral load results and discovering that they did not meet inclusion criteria because they were virally suppressed.

For the power analysis, from our prior work, we identified effect sizes from 0.64 to 1.0 for adherence and 0.55 to 0.91 for depression [16, 17]. We, therefore, powered the study at medium effects (0.50) for both ART adherence and depression outcomes. A sample size of 80 participants randomized per arm resulted in having at least 80% power to detect a 10% between‐group difference in adherence at 4 months. For depression, it was also powered (>80% power) to detect a 10% difference.

### 2.5 Randomization

To allow for stratified randomization (randomly rotating blocks of 6, 4 and 2) by whether or not a participant had started antidepressant medication, randomization occurred approximately 1 month after the initial baseline assessment. The randomization sequence was generated by a computerized table in the data management software (REDCap [29]) with two strata (site and antidepressant status). Study arm was only revealed after entering relevant information.

### 2.6 Blinding

After assignment to condition, the clinical assessor, blinded to study arm, administered the measures via interview, including the HAMD, MINI and CES‐D.

### 2.7 Statistical methods

Please see the online appendix (Appendix S1) for details of the analytic plan. Longitudinal models were employed whenever there were three or more assessment points. In the acute outcome period, a significant interaction indicates differential improvement across the two conditions. In the follow‐up period, interaction terms indicate differences in change after the treatment was over (so either significantly greater improvement or significantly stronger worsening). Main effects for study condition, in the absence of interactions in the follow‐up period would indicate continued differences between the two conditions.

### 2.8 Role of funding source

The study sponsor did not have a role in the design of the study, collection of data, analysis, interpretation, writing of the report or the decision to submit the paper for publication.

## 3 RESULTS

See Table 1 for demographics of the sample, Table 2 for estimated means for the outcomes at the specific timepoints and Figure 1 for participant flow. Of the 80 participants randomized to CBT‐AD, 86% (*n* = 69) attended all eight sessions.

**Table 1:** Demographics and descriptive statistics

Demographics	*N* (%)	Descriptive statistics	M (SD) Range
Gender			
Man	48 (29.8%)	Monthly income before tax (USD)	$133.09 ($164.58) $0–$1,097.87
Woman	114 (69.6%)		
Transgender man	1 (0.6%)	HAM‐D (at baseline)	24.74 (6.64) 8–38
Race		CES‐D (at baseline)	35.36 (11.50) 8–60
Black	160 (99.4%)	Raw viral load (at baseline)	81,374.79 (180,357.57) 0–1,241,488
Coloured	1 (0.6%)	Log transformed viral load (at baseline)	4.13 (0.98) 0–6·09
Education		Absolute CD4 cell count (at baseline)	213.91 (182.25) 3–1031
Grade 6 or below	15 (9.3%)	Uncensored adherence at randomization	62.17% (35.53%) 0–100%
Grade 7	14 (8.7%)	Censored adherence at randomization	67.77% (34.10%) 0–100%
Grade 8	16 (9.9%)		
Grade 9	21 (13%)		
Grade 10	28 (17.4%)		
Grade 11	47 (29.2%)		
Grade 12	15 (9.3%)		
Vocational training	2 (1.2%)		
University	3 (1.9%)		
Treatment regimen (at baseline)			
Re‐initiated on first line	82 (50.9%)		
Second line	79 (49.1%)		
Prescribed antidepressant medication (at randomization)			
CBT‐AD	3 (3.75%)		
ETAU	7 (8.64%)		

*Note*. In the race category, the term “Coloured” refers to a racial category during the apartheid era and remains relevant in describing present health disparities in South Africa.

Abbreviations: CBT‐AD, cognitive behavioral therapy for adherence and depression; CES‐D, Center for Epidemiologic Studies Depression Scale; ETAU, enhanced treatment as usual; HAM‐D, Hamilton Depression Rating Scale; M, mean; SD, standard deviation; USD, Unites States Dollars.

**Table 2:** Adjusted means for analyses across discrete timepoints and dichotomous viral load at 12‐month follow‐up by study condition, mean (standard error) or *N* (%)

	CBT‐AD		ETAU	
	Baseline	4 Months	Baseline	4 Months
HAM‐D	23.75 (0.83)	8.02 (0.92)	25.76 (0.83)	14.90 (0.92)
CES‐D	33.61 (1.42)	7.42 (1.56)	36.86 (1.41)	19.57 (1.56)
Log viral load	3.99 (0.18)	2.65 (0.14)	4.34 (0.18)	3.00 (0.14)

	CBT‐AD			ETAU		
	4 Months	8 Months	12 Months	4 Months	8 Months	12 Months
HAM‐D	9.04 (0.95)	8.69 (0.92)	7.51 (0.92)	14.67 (0.96)	14.33 (0.94)	13.15 (0.94)
CES‐D	9.47 (1.65)	8.29 (1.59)	6.20 (1.56)	18.92 (1.66)	17.75 (1.62)	15.66 (1.59)
Log viral load	3.32 (0.14)	3.21 (0.19)	3.44 (0.19)	3.67 (0.14)	3.56 (0.19)	3.79 (0.19)

Abbreviations: CBT‐AD, cognitive behavioral therapy for adherence and depression; CES‐D, Center for Epidemiologic Studies Depression Scale; ETAU, enhanced treatment as usual; HAM‐D, Hamilton Depression Rating Scale.

For the primary depression outcome, HAMD scores from baseline to 4 months, there was a significant interaction of time‐by‐condition, indicating that those in the CBT‐AD condition improved by an estimated 4.88 points (CI: –7.86, –1.87; *p* = 0.0016) more than those in ETAU. Superiority of CBT‐AD on acute treatment of depression was replicated with the self‐report CES‐D scores, indicating that CBT‐AD improved by an estimated 9.08 points (CI: –14.03, –4.12; *p* = 0.0004) more than ETAU during this time.

**Figure 2:**
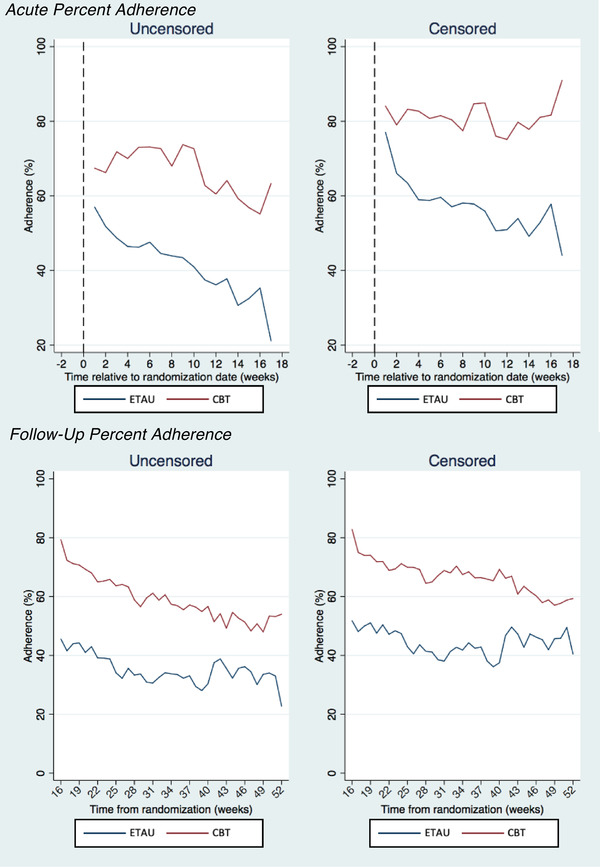
Acute (randomization to 4‐month follow‐up) and follow‐up (4‐ to 12‐month follow‐ups) percent adherence. *Note*. The treatment conditions are denoted on the bottom of each graph: enhanced treatment as usual (ETAU) and cognitive behavioral therapy for adherence and depression (CBT). The graphs on the right depict weekly percent adherence, censored for suspected non‐usage (1) starting at the time when a consecutive pattern of 0 openings began for participants who were lost to attrition, (2) days when the battery was dead and (3) for participants with a viral load as undetectable but their adherence was less than 80%. The graphs on the left depict the raw weekly percent adherence by condition over the same time periods.

For the primary adherence outcome, baseline to 4‐month (Figure 2), as hypothesized, there was a significant time by condition interaction (est = 1.61, CI: 0.64, 2.58; *p* = 0.001) during the acute treatment phase such that each week between randomization and 4 months, the CBT‐AD condition improved their adherence by approximately 1.61 percentage points more than ETAU per week. Accordingly, during this time, those receiving ETAU decreased adherence by approximately 1.48 percentage points per week (est = –1.48, CI: –21.2, –0.84), while the CBT‐AD condition showed a non‐significant weekly increase in adherence by approximately 0.13 percentage points (est = 0.13, CI: –0.60, 0.86; Figure 2). The sensitivity test censoring for potential Wisepill non‐usage revealed a similar pattern of results (interaction est = 1.59, CI: 0.83, 2.34; *p* < 0.001; ETAU significant decrease est = –1.37, CI: –1.92, –0.82, *p* < 0.001; CBT‐AD non‐significant increase per week est = 0.22, CI:–0.31, 0.75), showing superiority of the CBT‐AD condition.

For the depression in the follow‐up period, the CBT‐AD condition maintained superiority for lower depression over time on the HAMD. Accordingly, over the 4‐, 8‐ and 12‐month follow‐ups, they had an estimated 5.63 points lower scores (b = –5.63, CI: –7.90, –3.36; *p* < 0.001) over time than ETAU. There was a non‐significant trend for continued improvement across both conditions (b = –0.19, CI: –0.40, 0.14; *p* = 0.067), and the interaction of time and condition was, as hypothesized, not significant during the follow‐up period (revealing that the groups did not differ with respect to any changes after the intervention period ended/during the follow‐up). This pattern of results was generally replicated with the self‐report CESD indicator of depression, which showed that the CBT‐AD condition had, over follow‐up, an estimated 9.45 points lower scores (b = –9.45, CI: –13.28, –5.60; *p* < 0.001). However, the effect of time attained statistical significance, showing continued improvement in both conditions (b = –0.41, 95% CI: –0.78, –0.04; *p* = 0.030). As hypothesized, the interaction was not significant over the follow‐up period.

For follow‐up adherence, the CBT‐AD condition maintained superiority over the 4‐, 8‐ and 12‐month timepoints (Figure 2) as evidenced by the significant main effect showing approximately 24 percentage points higher adherence than ETAU (est = 23.56, CI: 13.25, 33.88; *p* < 0.001) in the absence of a significant interaction or main effect for time. The sensitivity analysis, taking into account potential non‐usage, revealed a similar result (est = 22.56, CI: 13.24, 33.88, *p* < 0.001).

For HIV RNA viral load detectability at 12 months, those in the CBT‐AD condition had 2.51 greater odds of having an undetectable viral load compared to those in ETAU (aOR = 2.51, CI: 1.01, 6.66, *p* = 0.047). CD4, however, did not significantly differ at 12 months. At the 12‐month timepoint, 19 (32%) individuals in the CBT‐AD condition had an undetectable viral load compared to 11 (20%) individuals in the ETAU condition with an undetectable viral load.

**Figure 3:**
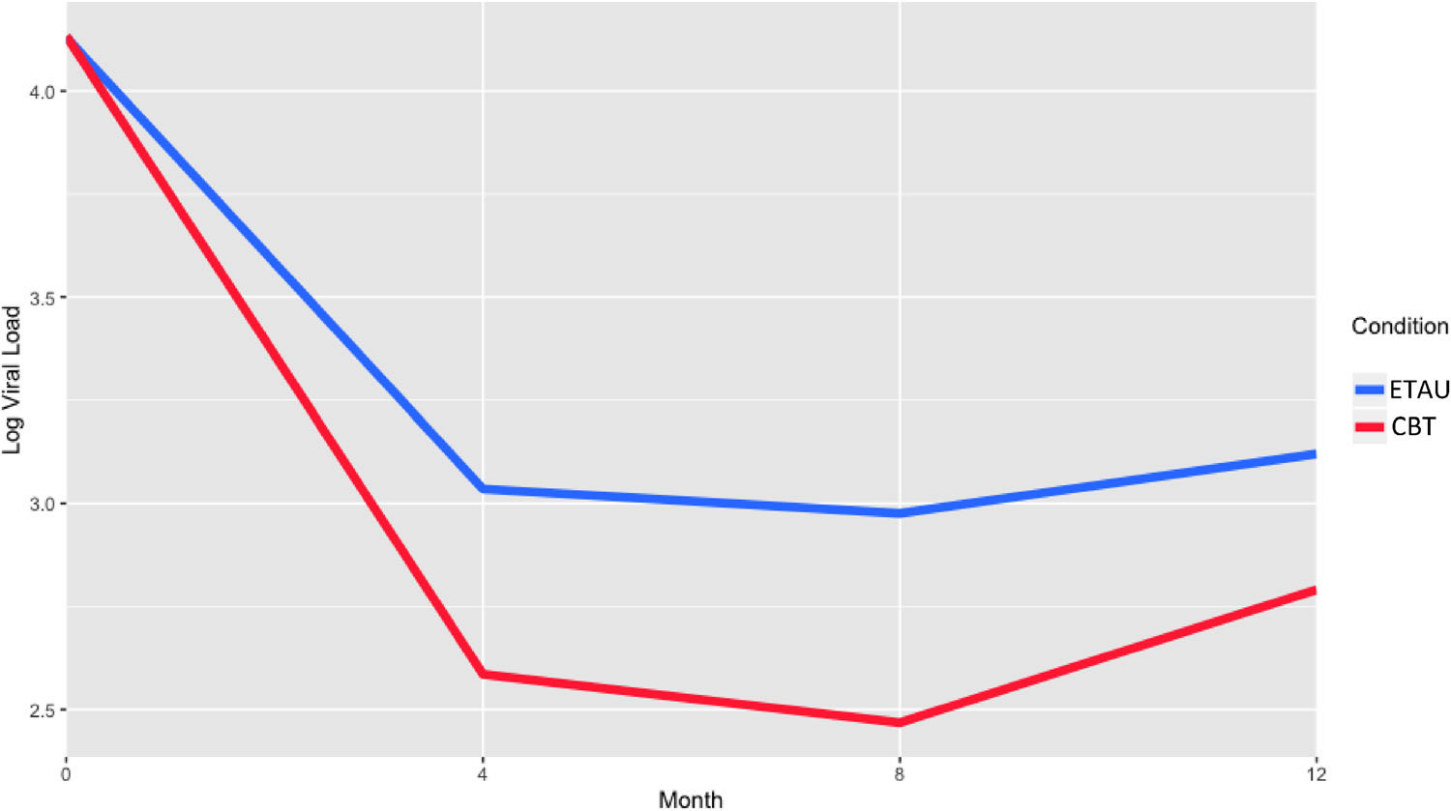
Log HIV viral load. *Note*. The treatment conditions are denoted on the right of the graph: enhanced treatment as usual (ETAU) and cognitive behavioral therapy for adherence and depression (CBT). Log transformed HIV viral load is depicted by condition.

The additional analyses of HIV RNA viral load showed superiority for the CBT‐AD condition as well. Those in CBT‐AD had a 3.54 greater odds of having an undetectable viral over the three follow‐up timepoints (OR = 3.54, CI: 1.59, 20.50, *p* = 0.038). When looking at log viral load continuously, those in CBT‐AD also had a greater decline than did those in ETAU in viral load from baseline to 4‐month, as evidenced by a significant interaction between condition and the period of time (b = –0.15, 95% CI: –0.29, –0.01, *p* = 0.042), and in this model, there was no significant interaction between condition and time over follow‐up (Figure 3). Analyses of CD4 over time did not show significant differences by study arm.

## 4 DISCUSSION

A task‐shared CBT‐AD intervention was effective in increasing adherence and decreasing depression over 12 months among PLWH with clinical depression. Given the shortage of mental health professionals in many low‐ and middle‐income countries, this study shows that it is possible to train nurses, a cadre of workers who are government‐funded and consistently situated in specialty (HIV) primary care clinics, in delivering CBT‐AD. This task‐shared approach may be scaled up to populations of individuals with other chronic conditions (including other infections or noncommunicable diseases). Accordingly, the burden of depression and its effects on broader morbidity and mortality could be significantly decreased. Further evidence for feasibility and acceptability is that 86% of patients assigned to CBT‐AD attended all eight sessions.

We found that using an objective indicator, Wisepill, adherence was superior in the CBT‐AD condition. The inclusion of an intervention to improve the key health behaviour of medication adherence into the treatment of depression adds to the literature that integrating treatment of depression with an intervention to improve health behaviour can be an effective strategy. This inclusion is in contrast to studies that only treat depression (with antidepressant therapy) in HIV but have not simultaneously addressed adherence whereby the depression improves and the adherence does not [13, 14]. These findings, in conjunction with findings showing effects for adherence interventions in general populations of individuals with HIV [3], suggest that using an evidenced‐based treatment of a mental health problem to “turn down the volume” of the mental health symptoms may allow for individuals to then seek optimal benefit from adherence counselling.

The large magnitude of the effect for Wisepill‐based adherence counselling speaks to the powerful effects on health behaviour change that can be achieved with an integrated intervention. Accordingly, the intervention here combines treating mental health distress with specific counselling for health behaviour change in a population that might otherwise be missed (those who fail to achieve viral suppression and have clinical depression) in ETAU interventions.

The secondary finding that the CBT‐AD condition had a higher percentage of patients with undetectable viral load at the 12‐month outcome, supported by the additional findings that CBT‐AD had higher percentages of patients with undetectable viral load across the follow‐ups, and that there was a greater decrease in continuous log viral load in the CBT‐AD condition, underscores the potential health impact and durability of the CBT‐AD intervention. Prior studies of CBT‐AD that did not select for individuals with a detectable viral load have generally found effects for depression and adherence, but not viral load [12, 16, 17]. The present study, which selected for those who had failed first‐ or second‐line treatment and had a viral load inclusion criterion, may have had greater power to find a viral load result than the prior trials. Future studies should follow patients longer to potentially examine distal effects of CD4, given the effects on adherence and viral load.

There are several limitations to note in the present study. First, Wisepill adherence, although it measures when the pillbox is opened and closed, does not measure whether the person actually ingested the medicine. This limitation is mitigated, however, by the viral load results and our sensitivity test. Second, although this was an effectiveness trial, implementing this intervention in a real‐world setting as conducted in the trial (tracking retention, reminder calls, Wisepill, etc.), as well as using clinic‐based staff and supervisors rather than study interventionists and supervisors, may require significant resources. Therefore, the cost‐effectiveness analysis that is currently underway for this trial will be important for policy implications. Relatedly, we hired the study nurse interventionist to be dedicated to delivering the intervention. Cost‐effectiveness and implementation science research is needed to determine the best ways of using existing clinic staff and freeing up time, and or adding additional nurse time to cover this potential work. Third, although there were improvements in viral load in those in the CBT‐AD arm compared to those in the ETAU arm, only approximately one‐third were at levels lower than detection at 12 months. The present study did not have stored samples for resistance testing to examine whether the marked improvements in adherence via Wisepill that did not result in undetectable viral load had to do with resistance to ART regimen. Fourth, the effect of booster sessions could not be examined because they were optional, and therefore, use of booster sessions may be confounded by indication.

## 5 CONCLUSIONS

The present study demonstrates the effectiveness of a task‐shared treatment of depression and adherence in individuals with HIV and clinical depression and uncontrolled HIV. The combination of behavioural (adherence), psychological (depression) and biomedical (viral load) outcomes in one treatment intervention for HIV in a setting where both HIV and clinical depression are prevalent, distressing and impairing conditions is of importance for global health approaches for managing these comorbid illnesses. CBT‐AD [25] integrates evidenced‐based strategies for treating depression with evidenced‐based counselling (Life‐Steps [19, 20]) for improving adherence. Integrating depression treatment while addressing an important health‐behaviour change target in the context of HIV, and finding a biomedical outcome, is an approach worthy of research and implementation in clinical settings. These effectiveness results support subsequent implementation science research to evaluate how to sustainably integrate this wholistic but potentially scalable approach into HIV care in South Africa.

## COMPETING INTEREST

Dr. Safren receives royalty statements from Oxford University Press, Guilford Publications and Springer/Humana Press for CBT treatment‐related material. All other authors have no interests to declare.

## DATA SAFETY MONITORING COMMITTEE

Drs. Kathleen Sikkema, Stacey Daughters and Crick Lund.

## AUTHORS' CONTRIBUTIONS

SS, CO, LA, JM and JJ conceptualized the program of research and much of the original design of the trial. LA also managed the study on the South Africa side, oversaw the intervention delivery, all study operations and supervised ground staff. JM also contributed to implementation components of the study throughout the trial period. JL managed the study from the U.S. side, including data management, and carrying out some of the statistical analyses under the supervision of SB. SB directed the analyses for all but the adherence outcomes. NM conducted the analyses for the adherence outcomes and managed the Wisepill adherence data. JS consulted on the cultural adaptation of the study as well as various design issues throughout the trial period. AK was part of the investigator team and contributed expertise on study design and cultural components as the trial went along. SS also wrote the first draft of the paper, with LA and JM helping with the introduction, and JL the figures and references. All authors edited, reviewed and approved the final manuscript.

## FUNDING

Funding for this project came from a National Institute of Mental Health grant R01MH103770. Some of the author time and resources for statistical consultation were also supported by grant 1P30MH116867.

## DISCLAIMER

The content is solely the responsibility of the authors and does not necessarily represent the official views of the National Institute of Mental Health or the National Institutes of Health.

## Supporting information


**Appendix S1**. Statistical analyses
**Appendix S2**. Details on translation process of depression measures
