# Supplementary material for: Treating depression and improving adherence in HIV care with task‐shared cognitive behavioural therapy in Khayelitsha, South Africa: a randomized controlled trial
Source: J Int AIDS Soc. 2021 Oct 28;24(10):e25823. doi: 10.1002/jia2.25823 (PMC8552453; doi:10.1002/jia2.25823)
Supplement: Supplementary file 1 — Appendix S1. Statistical analyses Appendix S2. Details on translation process of depression measures [file JIA2-24-e25823-s001.docx]

**APPENDIX 1 – STATISTICAL ANALYSES**

**Acute outcome**: For the Wisepill adherence, we analyzed weekly data longitudinally, both over the acute and over the follow up periods. This allowed for analysis of weekly data, and we used a generalized estimating equation model (GEE; uses all available data) with robust standard errors. To account for possible Wisepill non-usage (in the presence of actual ART adherence), we also report a sensitivity test whereby we censored weekly scores for suspected non-usage 1) starting at the time when a consecutive pattern of 0 openings began for participants who were lost to attrition, 2) days when the battery was dead, and 3) for participants with a viral load as LDL but their adherence was less than 80%.

To assess HAM-D (and replicated with self-reported CESD scores from baseline to post-intervention at 4-months we used linear mixed-effect models with maximum likelihood estimation for missing data, also using all available data and following intent-to-treat principles. Accordingly, for the acute outcomes, a significant effect for the interaction would indicate superiority of one condition over the other due to differential improvement from baseline.

**Follow up:** For Wisepill adherence, the same longitudinal approach (GEE) for the acute outcome was employed out to 52 weeks of data collection (the 12-month assessment); and with a similar approach to a sensitivity test for non-usage. For follow-up depression scores, we used multilevel modeling with the 4-, 8-, and 12-month data for all study outcomes. For these models, a non-statistically significant difference for the interaction term would indicate that the intervention condition maintained its gains over the comparison group, in that any changes post-treatment would be similar across the two conditions. Main effects for condition would further indicate superiority of CBT-AD in the follow-up period.

A-priori secondary analyses of biomedical outcomes were to compare the two study arms at the 12-month assessment for proportion of patients with detectable/undetectable HIV RNA viral load and for continuous CD4. For analysis of detectable versus undetectable HIV RNA viral load, we used Firth logistic regression, which is a penalized likelihood estimation method to correct for small sample bias[30] and allows for adjusted models*.* For CD4 cell count at 12-month we used multiple linear regression. Analyses were adjusted for baseline values (log HIV RNA viral load or CD4 cell number, respectively) and whether participants re-initiated first-line treatment or started second line regimen (binary).

As the biomedical outcomes data were available at all timepoints, longitudinal analyses of these outcomes were also conducted. For HIV RNA viral load we examined the proportion of patients with LDL viral load across 4-, 8-, and 12-month follow-ups using generalized linear mixed-effects modeling with maximum likelihood estimation. For these analyses, the same covariates were used (regimen and baseline log viral load). For continuous log viral load across all time points, we used a piecewise model with maximum likelihood estimation with the time from baseline to the 4-month follow-up as one piece and the time from 4- to 12-month follow-up as the second piece. For CD4 cell count across all time points, we used linear mixed-effects models with maximum likelihood estimation to examine CD4 as a continuous outcome.

**Missing data**: The generalized linear mixed model uses all observed timepoints for each patient when estimating model parameters. Any missing observations for the dependent variable are assumed missing at random adjusting for covariates and previously observed viral load. Maximum likelihood handling of missing data is equivalent to multiple imputation in this case^2^.

1. Heinze G, Schemper M. A solution to the problem of separation in logistic regression. 2002;11.
2. Allision, PD. Handling missing data by maximum likelihood. SAS Global Forum, 2005 <https://statisticalhorizons.com/wp-content/uploads/MissingDataByML.pdf>. Accessed 7/18/2021.

**APPENDIX 2 – Details on translation process of depression measures.**

The measures were translated using Brislin’s (1970) classic back-translation method^1^. Back-translation is a well-known method used to maintain equivalence when translating from source to target language. The measures were translated into isiXhosa, back-translated into English by an independent research assistant and checked by a third independent bilingual translator. When major discrepancies were evident, another round of translation and back-translation were performed by indepedent research assistants. This continued until no discrepancies remained. Final translations were reviewed by an isiXhosa speaking mental health specialist.

The CES-D has been validated in South Africa in PLWH ^2^. The HAM-D has been used in multiple antidepressant trials in South Africa^3, 4^. The MINI has been used as a gold standard clinical diagnostic interview in many studies in South Africa including in PLWH ^5^.

^1^Brislin RW (1970) Back-translation for crosscultural research. Journal of Cross-Cultural Psychology 1: 185–216.

^2^Myer L, Smit J, Roux LL, Parker S, Stein DJ, Seedat S. Common mental disorders among HIV-infected individuals in South Africa: prevalence, predictors, and validation of brief psychiatric rating scales. AIDS Patient Care STDS. 2008;22(2):147–58

^3^Kennedy SH and Emsley R (2006) Placebocontrolled trial of agomelatine in the treatment of major depressive disorder. European Neuropsychopharmacology 16(2): 93–100.

^4^Gagiano CA, Muller FG, Berk M, Joubert PM, Brown RG, Schall R. Moclobemide twice daily in the treatment of major depressive episode: a double-blind, multicenter comparison with diferent three times daily dosage schedules. J Clin Psychopharmacol. 1995;15(4):4S–9S. 25.

^5^Olley, B.O., Seedat, S., Neil, D.G., & Stein, D.J. (2004). Predictors of major depression in recently diagnosed patients with HIV/AIDS in South Africa. AIDS Patient Care and STDs, 18(8), 481487. Olley, B.O., Seedat, S., & Stein, D.J. (2006). Persistence of psychiatric disorders in a cohort of HIV/AIDS patients in South Africa: A 6-month follow-up study. Journal of Psychosomatic Research, 61(4), 479484.
